# Assessment of Reproductive Performance and Husbandry Practices of Horro and Crossbred Cattle Under Smallholder in Sibu Sire District of East Wollega Zone, Oromia, Ethiopia

**DOI:** 10.1002/vms3.70119

**Published:** 2024-11-09

**Authors:** Abriham Kebede Deresa, Jiregna Dugassa Kitessa, Yobsan Tamiru Terefa

**Affiliations:** ^1^ Department of Veterinary Medicine, School of Veterinary Medicine Wollega University Addis Ababa Oromia Ethiopia; ^2^ Department of Veterinary Medicine, College of Veterinary Medicine Addis Ababa University Addis Ababa Oromia Ethiopia

**Keywords:** breeding, dairy cattle, reproductive performance, Sibu sire

## Abstract

**Purpose:**

The reproductive and production performance of dairy cows determines the profitability of a dairy farm.

**Method:**

A questionnaire survey was used to assess reproductive performance and husbandry practices by using 100 randomly selected animal owners.

**Result:**

Seventy‐nine percent of animal owners involved were men, ages between 30 and 60. Their education was as follows: 26% were illiterate, 67% in primary school and 7% in secondary school. Ninety‐five percent of them managed their animals extensively and used natural mating and breeding practices (75%). AFS in months were 46.83 ± 0.65 in Ada‐Buke Warego, 46.00 ± 1.36 in Hagalo‐Tulam, 47.08 ± 0.99 in Dicho Aba‐Garmama and 47.29 ± 0.51 in Waligalte. AFC in months were 56.68 ± 0.97 in Ada‐Buke Warego, 58.00 ± 1.36 in Hagalo‐Tulam, 57.23 ± 1.41 in Dicho Aba‐Garmama and 56.24 ± 1.13 in Waligalte. ANSPCs were 1.85 ± 0.08 in Ada‐Buke Warego, 1.67 ± 0.14 in Hagalo‐Tulam, 1.54 ± 0.14 in Dicho Aba‐Garmama and 2.00 ± 0.10 in Waligalte. The mean length of CI in months was 30.88 ± 0.90 in Ada Buke Warego, 29.00 ± 1.68 in Hagalo‐Tulam, 28.62 ± 1.62 in Dicho Aba‐Garmama and 31.06 ± 1.04 in Waligalte. The average year of longevity was 9.22 ± 0.7 in Ada‐Buke Warego, 9.08 ± 0.8 in Hagalo‐Tulam, 9.31 ± 0.13 in Dicho Aba‐Garmama and 9.21 ± 0.8 in Waligalte, but there is no significant association (*p* ≥ 0.05). AFC in months was 48.00 ± 0.00 in local and 37.20 ± 1.30 in crossbreeds. ANSPC was 1.92 ± 0.06 in locals and 1.10 ± 0.10 in hybrids. AFC for Horro and crossbred dairy cattle were 57.73 ± 0.59 and 48.00 ± 0.00 months, respectively. The mean length of CI was 30.53 ± 0.67 and 29.40 ± 1.99 months in natives and crossbreeds, respectively. The average year of longevity was 9.22 ± 0.05 and 9.10 ± 0.10 years for local and hybrids, respectively. ANSPC for natural mating and AI were 1.92 ± 0.05 and 1.10 ± 0.10, respectively, with significant association (*p* < 0.05).

**Conclusion:**

The reproductive performance of dairy cows in the study area was low, which required the improvement of husbandry practices.

## Graphical abstract

The cross‐sectional study was used to obtain data on the reproductive performance of dairy cattle and their husbandry practices through a questionnaire survey and focal group discussion with animal owners. The total 100 respondents were animal owners, developmental agents (DAs) and veterinary workers. The reproductive performance of the animals and their husbandry practices were found to be lower compared to the other areas. Hence, dairy cattle traits and production constraints are essential to improving the reproductive performance of dairy cattle.

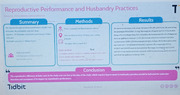

**Abbreviations**
AFC: age at first calving
AFS: age at first service
ANSPC: average number of procedures per conception
CI: calving interval
CSA: Central Statistical Agency
DA: development agent
ILCA: International Breeding Centre for Africa
MoARD: Ministry of Agriculture and Rural Development
NSC: number of services per conception
PAs: peasant associations
SSDBLF: Sibu Sire District Bureau of Livestock Resource Development and Fisheries

## 1 Introduction

Ethiopia is believed to have the largest livestock population in Africa, with 59,486,667 head of cattle (CSA 2017). Of this total cattle population, females represent approximately 55.5%, and the remaining 44.5% are males. Of the total milk produced in Ethiopia, 83% comes from cattle, while the rest comes from goats and camels (MoARD 2007). This is lower than the reports of CSA (2011) and CSA (2017); cows contribute about 95% and 94.6% of the total annual milk produced compared to other livestock species, respectively.

Despite the largest cattle population in Ethiopia, their production and reproductive performance are very low (Yoseph et al. 2003; Belay et al. 2012; Melku, Kefyalew, and Solomon 2016). Similarly, the overall reproductive performance of dairy cows has been found to be below the optimum values acceptable for profitable milk production in various parts of Ethiopia (Niraj, Kbron, and Abraha 2014; Nibret and Tadele 2014). Reasons for low performance in dairy cattle are likely due to genetic and environmental factors (Amin, Mahmoudand, and Megahed 2017; Amin et al. 2019), such as feed shortages, poor management, inadequate access to pasture, disease (Amin et al. 2021; Amin et al. 2022; Amin, Mahmoud, et al. 2023; Amin, Gamal, et al. 2023) and the absence of husbandry management (i.e., accurate oestrus detection and timely insemination). These factors significantly contributed to long open days (postpartum anaesthesia) (i.e., > 60 days after calving), late age at first calving (AFC), long interval between calvings, short lactation period and low milk production (Lobago et al. 2007).

The reproductive performance of breeding females may be the single most important factor underlying a sustainable milk production system and influencing the performance of dairy cattle. The size of the calf crop is important for the replacement of the herd, and milk production depends on the reproductive performance of the cow. The heritability of the reproductive trait is low; therefore, environmental factors, including managerial activities, play a significant role in the variability of the traits (Olori, Monwissen, and Veerkamp 2002). Reproductive traits such as age at first service, AFC, number of pregnancies, days open and calving interval (CI) are fundamental to profitable dairy farming (Mukasa‐Mugerewa 1989). Similarly, productive and reproductive performance traits make or break dairy farm enterprises because these traits are crucial in milk production (Cavestany and Galin 2001; Pursley, Kosorok, and Wiltbank 1997).

The profitability of dairy farms is determined by reproductive and production performance (LeBlanc 2007). It is the main factor that affects the overall productivity of milk production systems. This includes milk production efficiency, the number of calves produced per cow and lifetime milk production (Dejene 2014). Limited information is available regarding the reproductive and production performance of dairy cows in the tropics, specifically Ethiopia (Lobago et al. 2007). However, it is well known that indigenous and crossbred dairy cows under small farm conditions have poor reproductive performance. The specific factors that contribute to this poor reproductive performance have not been clearly identified, preventing the possibility of taking further steps to improve their performance. Therefore, the objective of the study was:

- To determine the reproductive performance of dairy cattle and identify management practices that may limit the reproductive performance of animals in Sibu Sire District, East Wollega Zone of Ethiopia.

## 2 Materials and Methods

### 2.1 Description of the Study Area

**FIGURE 1:**
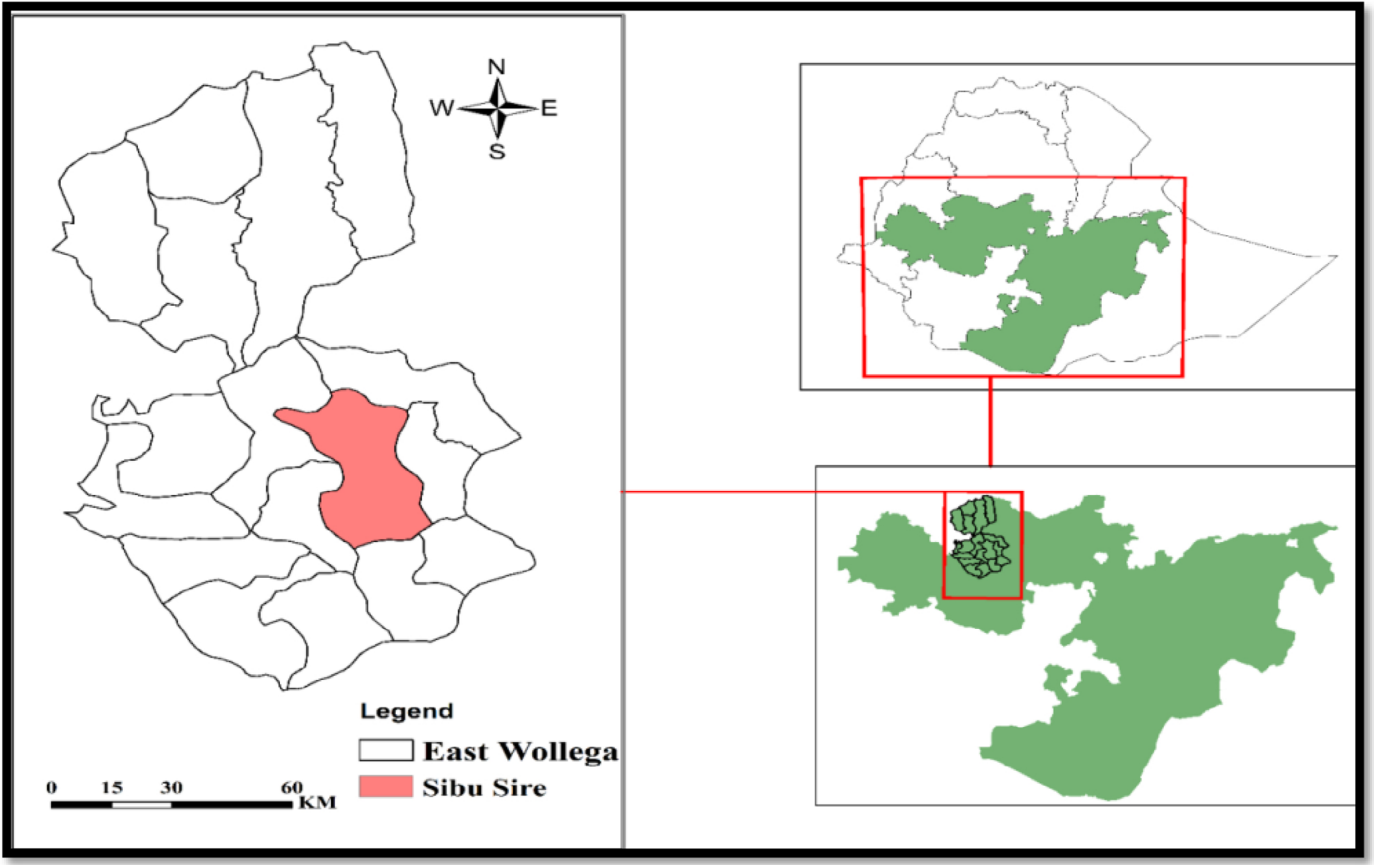
Map of the study area.

The study was conducted from December 2021 to April 2022 in Sibu Sire District, East Wollega Zone. Father Sibu is bound to Gobu Seyo in the east, Wayu Tuka in the west, Guto Gida and Gudeya Bila in the northwest and Wama Galo in the south (Figure 1). The district is divided into three different geographical areas with different proportions (i.e., 7.53% upland, 74.2% middle and 18.27% lower). The topographic features of the area are mostly rugged terrain, mountain ranges and plains. Many large and small rivers drained throughout the year are the reason for the rugged terrain of the area. The altitude varies from 1300 to 3020 m above sea level. As a result, the average annual temperature in the area is 15–20°C and the average annual rainfall is 1600–2000 mm. The total cattle population in this area is 172,941. Of this, cows 126,500, sheep 25,276, goats 32,773, mules 874, horses 180 and donkeys 8700 (SSDBLF 2008).

### 2.2 Study Population

The studied population consisted of animal owners in selected kebeles of the district. Both sexes and different age groups of animal owners who have at least one dairy animal were used for data collection.

### 2.3 Study Design

A cross‐sectional study was conducted to collect data using a questionnaire survey and focus group discussions with experienced dairy farmers, dairy experts and development agents (DAs).

### 2.4 Determining the Sample Size and Sampling Method

Four (4) peasant associations (PAs), namely Adabuke Warago, Hagalo Tulam, Dicho Aba Garmama and Waligalte, were purposefully selected from the district based on their proximity to veterinary clinics, availability of infrastructure and representativeness of the district. The sample size was determined using the formula recommended by Arsham (2002).

$$n=0.25/SE^{2}$$

where *n* is the sample size and SE is the expected standard error. A 5% standard error at the 0.05 precision level and a 95% confidence interval were used. Accordingly, a total of 100 individuals from the district who have at least one cow were interviewed. From each PA, the number of respondents involved varies according to their willingness to have animals. Accordingly, 41 from Ada Buke Warego, 12 from Hagalo Tulum, 13 from Dicho Aba Garmama and 34 from Waligalte were systematically selected.

### 2.5 Data Collection Methods

#### 2.5.1 Questionnaire Survey

A structured questionnaire was used to collect data from selected owners of dairy animals. The questionnaire was mainly focused on the socio‐economic characteristics of the household, the cattle breeding system, the challenges and limitations of cross‐breeding and management practices. Also, data on reproductive traits such as AFC, number of performances per conception, CI and maximum parity or longevity were considered indicators of reproductive performance (annex 1). It was translated into the local language before being distributed to or interviewed by animal owners.

#### 2.5.2 Focal Group Discussion

To strengthen the reliability of our assessment, a focus group discussion was conducted to obtain relevant data on the reproductive performance of dairy cattle and related constraints. This focus group consisted of DAs, veterinarians and animal owners (model farmers) who participated in the questionnaire. A checklist of different topics for focus group discussion was organised and presented to participants, and data was recorded for each topic. The cattle production system, the reproductive performance of dairy cows and factors that limit the reproductive performance of animals were discussed.

### 2.6 Statistical Data Analysis

Collected data were coded and entered into a Microsoft Excel 2007 spreadsheet. Data were analysed using SPSS software (version 2013) for descriptive statistics with cross‐tabulation for association used for questionnaire and focus group data on management practices. While reproductive parameters were analysed using one‐way ANOVA to compare the mean. For statistical analysis, the confidence level was kept at 95% and a *p* < 0.05 could be set for significance association.

## 3 Result

### 3.1 Respondent Demographics

According to the study, the majority of respondents in the district were men (79%); the rest were women (21%). The maximum and minimum age of respondents were 60 and 30 years, respectively. The educational background of the respondents was varied; 67% were at the primary school level, 26% were illiterate and 7% were at the secondary school level, as summarised in Table 1.

**TABLE 1:** Demographic description of the respondents of the research.

	No. of respondents per Kebeles				
Variables	Ada Buke Warego	Hagalo tulam	Dicho Aba Garmama	Waligalte	Total
Sex					
Male	32 (32%)	11 (11%)	9 (9%)	28 (28%)	79%
Female	9 (9%)	1 (1%)	4 (4%)	7 (7%)	21%
Age					
Minimum	30	30	30	30	
Maximum	55	60	50	50	
Education status					
Illiterate	8 (8%)	3 (3%)	7 (7%)	8 (7%)	26%
Primary school	30 (30%)	8 (8%)	5 (5%)	24 (24%)	67%
Secondary school	3 (3%)	1 (1%)	1 (1%)	2 (2%)	7%

### 3.2 Animal Breed and Purpose of Animal Husbandry

The results of the questionnaire show that the majority of respondents (90%) have local breeds, 10% have hybrids and keep animals for various purposes. Accordingly, 90% of the respondents keep animals for draft and milk, while 10% keep them for milk only, as shown in Table 2.

**TABLE 2:** Animal breed and purpose of keeping animals.

Variables	Frequency	Percentage
Breed		
Local	90	90%
Cross	10	10%
Purpose of keeping animals		
For milk	10	10%
Multi‐purpose	90	90%

### 3.3 Animal Husbandry and Husbandry Practices

Animal husbandry has a great influence on the production and reproductive performance of dairy cattle. Many pet owners (68%) do not provide supplementary feeding to their animals. Also, 67% of animal owners answered that they use river water as drinking water for their animals. The study also showed that 95% of pet owners raised their animals using extensive natural husbandry practices (75%). While the remaining animal owners raised their animals semi‐extensively using artificial insemination breeding practices. The study showed that there is a significant association (PV < 0.05) between husbandry practices and the reproductive performance of animals, as shown in Table 3.

**TABLE 3:** Summary of animal husbandry practice.

Variables	Frequency	Percentage	*p* value
Feeding			
Supplement feeding	32	32%	0.048
Non‐supplement feeding	68	68%	
Watering			
River water	67	67%	0.478
Tap water	33	33%	
Management			
Extensive	95	95%	0.037
Semi‐intensive	5	5%	
Breeding practice			
Natural mating	75	75%	0.0433
AI	10	10%	
Both AI and natural mating	15	15%	

### 3.4 Reproductive Performance of Animals

#### 3.4.1 Reproductive Performance Based on PA

The reproductive performance of dairy cattle varied depending on the PAs in the study area. PAs closer to the city had higher animal reproductive performance than those located in the remote area, but there is no significant association for all reproductive traits (*p* > 0.05), as shown in Table 4.

**TABLE 4:** Association of kebele and reproductive traits in dairy cattle.

	Kebeles (mean ± SE)				
Reproductive trait (in months)	Ada Buke Warego	Hagalo tulam	Dicho Aba Garmama	Wali Galte	*p* Value
Av. age at first mating	46.83 ± 0.65	46.00 ± 1.36	47.08 ± 0.99	47.29 ± 0.51	0.733
Av. age at first calving	56.68 ± 0.97	58.00 ± 1.36	57.23 ± 1.41	56.24 ± 1.13	0.862
Av. number of services per conception	1.85 ± 0.08	1.67 ± 0.14	1.54 ± 0.14	2.00 ± 0.10	0.064
Av. length of calving interval	30.88 ± 0.90	29.00 ± 1.68	28.62 ± 1.62	31.06 ± 1.04	0.484
Average year of longevity	9.22 ± 0.7	9.08 ± 0.8	9.31 ± 0.13	9.21 ± 0.8	0.597

Abbreviation: SE = standard error.

#### 3.4.2 Reproductive Performance Based on Animal Breed and Husbandry Method

The reproductive performance of dairy cattle varied depending on the type of animal breed and husbandry practices in the study area. Reproductive performance of animals was higher in crossbreeds and those using AI for breeding practices compared to local breed animals raised by natural mating. The study also showed that there was a significant association (*p *< 0.05) between animal breed, husbandry practices and animal reproductive performance except for CI and average parity number, as shown in Table 5.

**TABLE 5:** Association of animal breed and breeding practice with reproductive traits of dairy cattle.

	Animal breed			Breeding practice		
Reproductive trait	Local	Cross	*p* value	Natural mating	AI	*p* value
Av. age at first mating	48.00 ± 0.00	37.20 ± 1.30	0.00	48.00 ± 0.00	37.20 ± 1.18	0.00
Av. age at first calving	57.73 ± 0.59	48.00 ± 0.00	0.00	57.73 ± 0.66	48.00 ± 0.00	0.00
Av. number of services per conception	1.92 ± 0.06	1.10 ± 0.10	0.000	1.92 ± 0.05	1.10 ± 10	0.000
Average calving interval	30.53 ± 0.67	29.40 ± 1.99	0.573	30.53 ± 0.60	29.40 ± 1.72	0.57
Average number of longevity	9.22 ± 0.05	9.10 ± 0.10	0.373	9.22 ± 0.05	9.10 ± 0.10	0.373

#### 3.4.3 Reproductive Performance Based on Feeding Practice

The reproductive performance of dairy cattle varied depending on animal feeding practices in the study area. The reproductive performance of the animals varied depending on the PA, as the feeding practices differed between PAs. The study showed that there was a significant association (*p* < 0.05) between feeding practices and the reproductive performance of animals, except for CI and average parity number, as shown in Table 6.

**TABLE 6:** Association of Feeding and reproductive traits of dairy cattle.

Reproductive trait	Feeding		*p* value
	Supplement feeding	No supplement feeding	
Age at first mating	36.00 ± 0.00	47.38 ± 0.28	0.000
Age at first calving	48.00 ± 0.00	57.13 ± 0.62	0.003
Number of services per conception	2.00 ± 0.00	2.88 ± 0.06	0.004
Average calving interval	27.00 ± 2.90	30.56 ± 0.73	0.245
Average number of parity (in years)	9.00 ± 0.00	9.22 ± 0.04	0.297

#### 3.4.4 Reproductive Performance Based on Availability of Veterinary Services

The reproductive performance of dairy cattle varied with the availability of veterinary services in the study area as it relates to animal health. The availability of veterinary services affects the reproductive performance of the animal, but there is no significant relationship between them (*p* > 0.05), as shown in Table 7.

**TABLE 7:** Association between Veterinary service availability and reproductive traits of dairy cattle.

Reproductive trait (in months)	Veterinary service availability			*p* value
	No	Yes but far	Yes	
Age at first mating	48.00 ± 0.00	46.88 ± 0.44	46.20 ± 0.98	0.297
Age at first calving	58.25 ± 1.18	56.53 ± 0.92	56.30 ± 1.38	0.592
Number of services per conception	2.06 ± 0.13	1.80 ± 0.80	1.80 ± 0.12	0.250
Average calving interval	28.50 ± 1.37	31.13 ± 0.69	29.70 ± 1.88	0.246
Average number of parity (in years)	9.19 ± 0.11	9.25 ± 0.06	9.10 ± 0.06	0.353

## 4 Discussions

The current study showed that the educational attainment of the majority of respondents was low and ranged between illiteracy and elementary school. This is in line with a study conducted in different parts of the country that reported low levels of household education (Asaminew and Eyasu 2009; Nibret and Tadele 2014). This low level of education among animal owners may lead to poor animal husbandry with low adoption of dairy innovations, for example, forage cultivation, techniques of breeding and modern breeding of dairy cows in the study area.

The majority of respondents (90%) have cattle of the local breed (Horro breed) and keep the animal for multiple purposes (95%), mostly without supplemental feeding (68%). They kept their animals extensively (95%) and used natural mating practices (75%); 15% used both natural mating and AI, while only 10% used AI as a breeding practice. However, artificial intelligence is being used to reduce the number of conception procedures, limit disease transmission and improve animal husbandry, according to the study.

Reproductive performance based on kebeles (Ada Buke Warego, Hagalo Tulam, Dicho Aba Garmama and Wali Galte) mean age at first use was (46.83 ± 0.65, 46.00 ± 1.36, 47.08 ± 0.99 and 47.29 ± 0.51), average AFC 56.68 ± 0.97, 58.00 ± 1.36, 57.23 ± 1.41 and 56.24 ± 1.13) months, average number of procedures per conception (1.85 ± 0.08, 1.67 ± 0.14, 1.54 ± 0.14 and 2,000.0.0.0 ± average length of labour) 30.88 ± 0.90, 29, 00 ± 1.68, 28.62 ± 1.62 and 31.06 ± 1.04) months, average year of longevity (9.22 ± 0.7, 9.08 ± 0.8 and 9 ± 0.13 ± 9.31, 9.31 ± 0.13 and 9.21), but no significant association (*p* > 0.05). The finding was consistent with the finding of Mukasa‐Mugerewa (1989). This may be due to differences in climate suitability, availability of feed and animal owners' awareness of animal husbandry.

The reproductive performance of breeds (local and crossbred) had an average AFC (48.00 ± 0.00 and 37.20 ± 1.30) months, an average number of performances per conception (1.92 ± 0.06 and 1.10 ± 0.10), an average AFC for local and crossbred dairy cattle (57.73 ± 0.59 and 48.00 ± 0.00) months, average length of CI (30.53 ± 0.67 and 29.40 ± 1.99) months and an average year of longevity (9.22 ± 0.05 and 9.10 ± 0.00). The mean number of procedures per conception for natural mating and AI was (1.92 ± 0.05 and 1.10 ± 10) with a significant association (*p* < 0.05). The study showed that the reproductive performance of dairy cattle was better in the cross compared to the local (Horro) breed. This finding was consistent with Gillah, Kifaro, and Madsen (2012), who reported that crossbreeding improves the reproductive performance of dairy cattle. Reproductive performance based on feeding (supplemented and non‐supplemented) were mean age at first use (36.00 ± 0.00 and 47.38 ± 0.28) months, mean AFC (48.00 ± 0.00 and 57.13 ± 0.62) months, average number of services per conception (NSC) (2.00 ± 0.00 and 2.88 ± 0.06), average length of CI (27.00 ± 2.90 and 30.56 ± 0.73) months and average year of longevity (9.00 ± 0.00 and 9.22 ± 0.04), respectively, with significant association (*p* < 0.05). This finding was consistent with the finding of Belay et al. (2012). A relatively longer CI could indicate environmental factors (i.e., poor husbandry practices, poor husbandry management and disease), as reported by Jiregna, Abraham, and Yobsan (2021). Availability of veterinary services affects animal reproductive performance, which is a similar report of Jiregna, Abraham, and Yobsan (2021). As veterinary services, it is directly related to the protection of animal health problems that affect their reproductive performance.

## 5 Conclusion and Recommendations

The results of this study showed that the reproductive performance of local and crossbred cows was low with respect to breeds. Reproductive performance identified in dairy cattle in the study area was late AFC, long CI and high NSC. In addition, the main constraints on the reproductive performance of cattle in the study area are lack of fodder, repeated breeding, diseases, lack of veterinary services, lack of awareness of artificial insemination time and long distance for AI service. Dairy cattle traits and production constraints are essential to improving the reproductive performance of dairy cattle. Based on the above conclusion, the following recommendations were proposed:

- Animal husbandry should be improved to increase their reproductive performance.
- Provision of health services should be available to address health problems in the area.
- Appropriate selection of breeds for the crossbreeding program based on their performance.
- The level of education of the owner has an effect on the management of animals; therefore, the animal owner should be aware of animal management.

## Author Contributions

**Abriham Kebede Deresa**: conceptualisation, data curation, formal analysis, investigation, methodology, project administration. **Jiregna Dugassa Kitessa**: investigation, resources, software, visualisation, writing–review and editing. **Yobsan Tamiru Terefa**: formal analysis, investigation, methodology, validation, writing–original draft, writing–review and editing.

## Ethics Statement

Ethical approval for this study was obtained from the Research Ethics Review Board of the School of Veterinary Medicine, Wollega University, according to certificates Ref. No. VMERC78/03/2021. The study method was a questionnaire survey without the participation of animals; however, ethical considerations were reliable for animal owners. After ethical approval, all activities were carried out in accordance with the ethical guidelines of the School of Veterinary Medicine.

## Conflicts of Interest

The authors read and agreed on there is no conflict of idea on publishing this manuscript.

### Peer Review

The peer review history for this article is available at https://publons.com/publon/10.1002/vms3.70119.

## Data Availability

All peer‐reviewed data and questionnaires used for the study were summarized and included in this article. Data used for analysis can be obtained from the corresponding author if needed.
